# Combining Anatomical, Clinical, and Physiological Signs in Confirming Correct Tracheal Tube Placement: The Value in “Seeing (the Tube) Is Believing (in Its Position)”

**DOI:** 10.1213/ANE.0000000000007274

**Published:** 2025-01-10

**Authors:** Kariem El-Boghdadly, Jaideep J. Pandit

**Affiliations:** From the * Department of Anaesthesia, Guy’s and St Thomas’ NHS Foundation Trust, London, United Kingdom; † King’s College, London, United Kingdom; ‡ University of Oxford, Oxford, United Kingdom; § Nuffield Department of Anaesthetics, Oxford University Hospitals NHS, Oxford, United Kingdom.

**See Article, page 280**

In this issue of the journal, Markham et al report the findings of a study that challenged some reviewers, and which some readers may find troubling, if not difficult to believe. In a randomized crossover trial of patients with potentially difficult airways, the authors avoided intubating the trachea after induction of anesthesia and instead, deliberately placed the tracheal tube tip in the pharynx (TTIP) and compared the efficacy of ventilating the lungs through this with mask ventilation.^1^ The TTIP technique involves blindly inserting a tracheal tube to a target depth into the oropharynx, hoping for the tip of the tube to sit somewhere above the glottis, then sealing the mouth around the tube with 2 hands followed by ventilation. The theory is that in patients with difficult airway management and in whom facemask ventilation is difficult, blindly placing a tracheal tube into the pharynx may allow sufficient ventilation to salvage a critical scenario. Perhaps merely a “poor man’s supraglottic airway,”^2^ the technique has previously been described in various settings,^3^ but this study by Markham et al is the first to attempt rigorously to establish its efficacy in patients at risk of difficult airway management. Of the 136 patients in their final analysis, successful ventilation (their definition discussed below) occurred in 93% with the TTIP technique compared with 85% with facemask ventilation. The authors conclude that these were similar and that TTIP *could* be used to rescue difficult or failed facemask ventilation. Whether it should be used is the subject of this editorial.

## LIMITATIONS OF MARKHAM ET AL’S STUDY

Before commenting on the implications of this study, there are some limitations (many of which the authors acknowledge). Their definition of successful ventilation was liberal, requiring only end-tidal CO_2_ and expired tidal volumes for only 3 breaths; that is, there is no evidence that TTIP can be used to sustain oxygenation or ventilation. Indeed, it is notable that tidal volumes achieved with mask ventilation were significantly greater (by ~100 ml) than with TTIP. This is despite their facemask ventilation not being fully optimized by using an oropharyngeal airway, head-up positioning, or ensuring adequate neuromuscular blockade.^4^ Although they included patients with “potentially difficult airways,” patients who were judged to require awake intubation (ie, the truly difficult) were excluded. They do not report the laryngoscopy grade at intubation, but it is implied that in fact no difficulty was encountered. Importantly, we do not actually know where the tracheal tube tip sat, as depth measurements relied only on surface landmarks, but not on accurate anatomical assessment (eg, fiberoptic or radiologic confirmation). Finally, there was insufficient reporting of safety outcomes, such as gastric insufflation, pulmonary aspiration, or other assessments of trauma due to this technique. It is implied these did not arise, but gastric ultrasound could have been used as part of the comparison.

## A CLINICAL NICHE FOR TTIP?

Regardless of study limitations, readers may wonder if this technique has any niche in clinical practice. The authors suggest this would be in a “cannot intubate, cannot ventilate” (CICV) scenario, presumably when attempts at tracheal intubation have failed, as has supraglottic airway insertion. The practical difficulty here is that, when clinicians are faced with this scenario, the airway is likely to be traumatized, edematous, and anatomically distorted due to multiple attempts at instrumentation, so we do not know whether the TTIP technique will be viable. Additionally, we now know that the more steps that are introduced in crisis situations, the higher the cognitive load, and therefore the greater the challenges in decision-making and intervention.^5^ Furthermore, in a (CICV) scenario, rapid progression to emergency front-of-neck airway (eFONA) is widely seen as necessary,^6–9^ and thus delaying that definitive intervention by attempting this additional step may not necessarily be in the patient’s best interest.

## IMPLICATIONS FOR THE SCIENCE OF DIFFICULT AIRWAY MANAGEMENT

Beyond clinical practice issues, the results of Markham et al have implications for the wider debates around how best to confirm tracheal intubation (ie, exclude esophageal intubation), in particular, about the proper role and interpretation of capnography. The original Royal College of Anaesthetists (RCOA, UK) “*no trace = wrong place*” campaign emphasized that absence of a CO_2_ trace indicated that the tracheal tube was misplaced.^10^ To this, Pandit observed that “no trace, wrong place” should not be misinterpreted to mean that presence of a trace confirms correct tracheal intubation.^11^ The data of Markham et al appear to confirm that argument. Their tube tip in fact appears to lie in or at the top of the esophagus, and it seems it is the Murphy eye, or collateral flow of air into the trachea that facilitates end-tidal CO_2_ and oxygenation (see Figure 3A of their paper). In other words, their data importantly remind us that whereas absence of a CO_2_ trace strongly implies a misplaced tube, the presence of end-tidal CO_2_ cannot be regarded as assuredly confirming correct tube placement.

Although Markham et al only elicited a capnography trace for 3 breaths, in principle this result could be seen as challenging views expressed more recently by an international expert group (the Project for Universal Management of the Airway, PUMA), which sought to extend the RCOA guidance. The PUMA argued that end-tidal CO_2_ sustained over 7 breaths with a value >7.5 mm Hg (1 kPa) excluded esophageal intubation (ie, confirmed tracheal intubation).^12^ These recommendations have been criticized on several grounds: (a) the guidelines make the mistake of assuming that since absence of CO_2_ is a sign of misplaced tube, then the presence of CO_2_ confirms correct tube placement (this is false logic of “denying the antecedent”); (b) there is no evidence for a specific 1 kPa threshold; (c) the choice of exactly 7 breaths is insufficiently justified (ie, why not 6 or 8?); and (d) there are situations when correctly sited tubes may not meet PUMA criteria and therefore might end up being removed with adverse consequences.^13^ Critics argue that instead, correct tube placement should combine contextual clinical signs along with capnography, and not capnography alone. The results of Markham et al are further potential evidence that capnography alone might be insufficient to confirm tracheal intubation.

This then raises the question of how we can add further certainty to confirming tracheal tube placement. The Difficult Airway Society (DAS) has recommended a “two-point check” (capnography *and* visible tube in the trachea) before induction of anesthesia after awake or sedated tracheal intubation.^14^ The principle is that relying on just one variable (capnography alone or visualization alone) might reduce the confidence in the position of a tracheal tube, and therefore, combining two validated variables increases safety. If this logic and recommendation is valid in the awake tracheal intubation setting then we believe this concept should be extended to all tracheal intubations, not just those in patients whose airways are difficult.

We can therefore classify the signs of correct tube placement as 3: clinical (chest wall movement, breath sounds, misting of tracheal tube), physiological (end-tidal CO_2_ waveform), and anatomical (visualization of tube between vocal cords or within trachea). Where each of these is aligned, there is no dilemma: presence of all signs means correct placement; absence of all indicates misplacement. Dilemmas only arise with misalignment of these 3, which may be uncommon, but then clinical reasoning is necessary to diagnose the problem. Arguably, clinical signs are the most susceptible to individual interpretation but are virtually the only way quickly to diagnose other potentially life-threatening conditions like endobronchial intubation, pneumothorax, or bronchoconstriction. Absent chest movements or breath sounds despite end-tidal CO_2_ and anatomical confirmation of tube position may be signs of severe asthma. As Markham et al have now shown, chest movements and end-tidal CO_2_ are possible (at least for a very limited period of time) even with a misplaced tube that could at any time dangerously migrate into the esophagus. Conversely, a tube confirmed anatomically as intratracheal and good chest movements can be associated with absent CO_2_ if, for example, the monitor is faulty. This happened in a recent reported case: unfortunately, practitioners apparently followed PUMA guidance in prioritizing the capnography interpretation, and removed the tube with fatal consequences.^15^

Although it is difficult to say which of these (clinical, physiological, or anatomical) is the most “important,” there is a natural chronology that offers a ready logic tree to confirm correct tube placement. In sequence during intubation, first is the act of placing and hence visually confirming passage of the tube through the vocal cords. Soon after comes the action of ventilating the lungs, which gives rise to clinical signs. Contemporaneously, there appears end-tidal CO_2_. Performed rapidly and fluently, a mismatch of results from these should give cause for concern, as outlined in the Figure.

**Figure.**
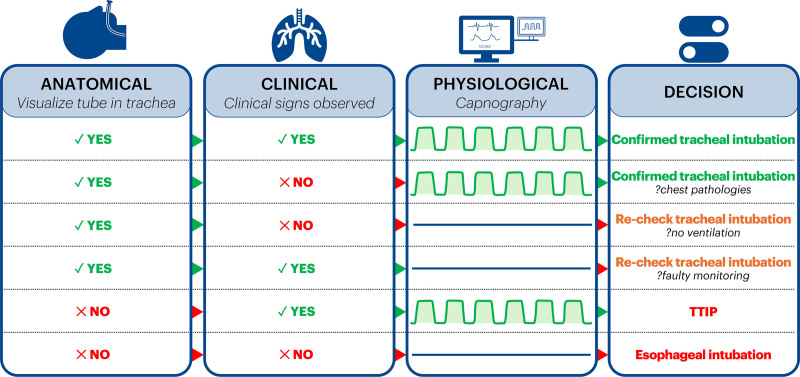
The 3 steps by which correct tracheal intubation is confirmed: anatomical (visualize tube in trachea); clinical (signs including chest movement, breath sounds); physiological (capnography). Each “yes” (green arrows) signifies that the result of that step is consistent with correct placement. Each “no” (red arrows) signifies that the result fails to confirm correct placement. TTIP = tube tip in pharynx. Chest pathologies include chest wall rigidity, bronchospasm, or any other pathology in which normal clinical signs may be obtunded. The situation of “no ventilation” might arise with absent or inadequate mechanical ventilation, leaks in the circuit, or large bronchopleural fistula. The conclusions to “recheck tracheal intubation” require the practitioner to take additional steps to check tube position after the primary intubation. For example, “glottic impersonation” is described in which blanched lateral aspects of the esophagus can be mistaken for the vocal cords at the primary intubation.^14^ Rechecking might include videolaryngoscopic, bronchoscopic or ultrasound confirmation of tube position.

The utility of the Figure is first, to show that none of the signs (anatomical, clinical, or physiological) is by itself “pathognomic” of correct or incorrect tube placement. In this view, we differ from PUMA guidance, which decidedly argues that capnogaphy trumps everything else. The PUMA team substantiated their argument by a Bayesian analysis that assessed the low likelihood of several clinical signs being able individually to exclude esophageal intubation as compared with capnography.^15,16^ Notably, in this study Hansel et al did not assess the value of several clinical signs collectively, or of direct visual confirmation of tube placement. Our Figure emphasizes the binary outcome of such visualization, in contrast to the interpretation of all other signs. In other words, if anatomically the tube is seen to be misplaced then the situation cannot be accepted, regardless of any other sign including capnography which, as Markham et al show, can arise even with a tube lying outside the trachea.

This leads to the important question of what to do if the tube has *not* been seen to pass the vocal cords, as may be the case in “blind” intubation such as with Grade 3 or 4 laryngoscopy? Hitherto, practitioners have just continued with anesthesia/ventilation as long as clinical and physiological signs are satisfactory (chest movements, end-tidal CO_2_, oxygenation). However, we now advise that, even if capnography and clinical signs are confirmatory, the anatomical position of the tube should *always* be promptly rechecked, with videolaryngoscopy, fiberoptic bronchoscopy, ultrasound, or another form of practical imaging modality (after all, this could be inadvertent TTIP).^17^ The delay with which this is done will depend on context (clinical instability may require other interventions to be taken first), but especially in the context of any desaturation, this mandatory anatomical confirmation should not be unduly postponed. The Figure properly sets the primacy of anatomical confirmation in relation to the other clinical and physiological signs.

## CONCLUSIONS

We find it difficult to recommend TTIP as something to be embedded in airway management guidelines. In larger studies, we suspect the consequences of “failed TTIP” (esophageal tube migration, gastric insufflation, and aspiration, pharyngeal trauma) will become all too evident. At best, the findings of Markham et al offer some small reassurance to practitioners inexperienced in eFONA to attempt one last-ditch technique to rescue a difficult situation, but no more. The provocative results of Markham et al have implications for some contemporary thinking about airway management. Arguments that place capnography on a pedestal above other signs are superseded in our view by the value of combining more variables for confirming tracheal intubation in a rational way (Figure). For the clinician, safety is best achieved when, as with existing guidance for awake intubation, the correct anatomical position of the tube is always confirmed (ideally, and as a 2-person check):^18^ seeing the tube is indeed believing in its correct position.

## DISCLOSURES

**Conflicts of Interest:** K. El-Boghdadly and his institution have received funding from Fisher and Paykel, PAION, GE Medical, Cardinal Health, and Edwards Lifesciences. J. J. Pandit is Editor-in-Chief of *Anesthesia & Analgesia*, and was not involved in the handling of this manuscript. No other authors declared Conflicts of Interest. **Funding:** None. **This manuscript was handled by:** Narasimhan Jagannathan, MD, MBA.
